# Integrated Radiology–Biochemistry Diagnostic Flow Framework for Emergency Clinical Decision Support: A Simulation-Based Educational Model

**DOI:** 10.3390/tomography12020016

**Published:** 2026-01-27

**Authors:** Betül Tiryaki Baştuğ, Türkan Güney

**Affiliations:** 1 Department of Radiology, Faculty of Medicine, Bilecik Şeyh Edebali University, Bilecik 11000, Turkey; 2 Department of Biochemistry, Faculty of Medicine, Bilecik Şeyh Edebali University, Bilecik 11000, Turkey; turkan.guney@bilecik.edu.tr

**Keywords:** emergency radiology, decision support, structured reporting, diagnostic flow framework, synthetic cases, multimodal integration, radiology education, biochemistry integration

## Abstract

Background: Emergency radiology often demands rapid integration of clinical cues, biochemical markers, and imaging findings to support time-critical diagnostic reasoning. However, educational resources that explicitly structure this interdisciplinary integration particularly between radiology and laboratory medicine remain limited. Objective: Our objective was to develop an Integrated Radiology–Biochemistry Diagnostic Flow Framework as a simulation-based methodological proof-of-concept and to document its structure, logic pathways, and internal consistency across common emergency presentations. Methods: We designed an algorithmic framework combining (i) clinical triggers, (ii) targeted biochemical markers with predefined threshold and trajectory rules, (iii) imaging indication and modality selection (US/CTA/MRI/NCCT), and (iv) key radiologic patterns linked to escalation pathways. No patient data or human participants were included. Instead, forty fully synthetic emergency scenarios were generated to populate the framework and to examine logical completeness, branching coherence, and red-flag escalation routes. Results: The framework yielded scenario-specific diagnostic flowcharts that systematically connect biochemical escalation cues with imaging selection and expected imaging findings. The synthetic scenario library demonstrated consistent branching logic across conditions and enabled transparent visualization of imaging-centered decision pathways suitable for simulation-based teaching and structured case discussion. Conclusions: This study reports a reproducible methodological proof-of-concept framework and a synthetic emergency scenario library. Further learner-based studies are required to evaluate usability, perceived realism, and educational effectiveness in authentic training settings.

## Simple Summary

This study presents a simulation-based Integrated Radiology–Biochemistry Diagnostic Flow Framework developed to support emergency diagnostic reasoning in a structured and stepwise manner. The framework brings together clinical presentation, relevant laboratory markers (when applicable), imaging findings, and an explicit decision/escalation point within a single pathway. It was applied to 40 fully synthetic emergency scenarios representing different acute care domains. Within this controlled setting, the standardized structure allowed each case to be organized toward a predefined management-oriented endpoint and facilitated transparent linkage between biochemical and radiological reasoning. Because the scenarios were entirely synthetic and no learners or clinical workflows were evaluated, the framework is intended as an educational proof-of-concept. It may serve as a practical teaching tool for medical students and early trainees and provides a basis for future empirical evaluation in training environments.

## 1. Introduction

Rapid and accurate diagnostic decision-making is a cornerstone of emergency medicine, where clinicians must interpret clinical findings alongside laboratory data and imaging results under significant time pressure [1,2]. Emergency presentations such as mesenteric ischemia, small-bowel perforation, pulmonary embolism, pancreatitis, intracranial hemorrhage, and complicated ileus often require immediate the correlation of biochemical markers with radiological patterns to establish a timely diagnosis [3,4]. However, medical students and novice clinicians commonly face challenges in synthesizing these multimodal data streams, partly due to the fragmented nature of traditional medical education. Radiology and biochemistry are generally taught as separate disciplines, resulting in limited opportunities for structured integrative reasoning during acute clinical scenarios.

The separation of diagnostic disciplines in education contrasts sharply with the realities of emergency care, where decisions must be taken through a unified interpretation of complementary diagnostic modalities [5,6]. For example, elevated serum lactate may suggest ischemia, but its diagnostic value substantially increases when integrated with imaging findings such as decreased bowel wall enhancement or pneumatosis intestinalis. Similarly, markedly elevated D-dimer levels may prompt CT pulmonary angiography, while specific patterns of hyperamylasemia or leukocytosis guide the interpretation of CT findings in acute pancreatitis. Without an educational approach that explicitly demonstrates these relationships, learners may struggle to form accurate diagnostic hypotheses and recognize critical red flags across disciplines [7,8].

This need is further amplified by the rapid expansion of high-volume imaging in emergency departments and the parallel growth of laboratory-driven triage pathways. In real-world workflows, radiologic interpretation and biochemical markers are rarely considered in isolation; instead, clinicians continuously integrate imaging patterns with inflammatory indices, metabolic derangements, and organ function indicators to narrow differential diagnoses and prioritize life-threatening conditions. However, most existing educational materials present radiology and laboratory interpretation in parallel rather than within a single structured decision pathway, which may leave trainees without explicit guidance for multimodal integration during time-critical decision-making.

Simulation-based learning has gained prominence as a pedagogical method capable of addressing this gap. Simulation provides an interactive, learner-centered environment where complex scenarios can be deconstructed into manageable components, allowing students to develop structured diagnostic strategies in a safe, controlled, and repeatable setting [9,10]. Unlike retrospective case discussions, simulation permits immediate feedback, promotes active decision-making, and reflects the time-sensitive pressures of real clinical care. Importantly, simulation using synthetic cases avoids the need for patient data and therefore does not require ethical approval, making it an ideal approach for educational research and curriculum development.

Despite the proven benefits of simulation in medical training, there remains an absence of structured interdisciplinary diagnostic frameworks that integrate radiology with laboratory medicine for emergency presentations. Existing structured reporting templates in radiology do not incorporate biochemical results, while laboratory interpretation guides typically omit imaging considerations. This disconnect may contribute to cognitive overload, fragmented reasoning, and variability in clinical decision-making—particularly in acute care settings where prompt integration of findings is essential. In emergency workflows, imaging often serves as the primary diagnostic anchor, whereas laboratory markers refine probability and urgency; therefore, an integrative educational model should remain imaging-centered while explicitly incorporating biochemical escalation cues.

To address this gap, we developed an Integrated Radiology–Biochemistry Diagnostic Flow Framework, a tool designed to guide learners through the sequential synthesis of laboratory markers and radiological findings in emergency scenarios. The framework visualizes the progression from initial clinical suspicion to the integration of laboratory abnormalities and imaging features, culminating in a structured diagnostic conclusion. By placing radiology and biochemistry within a unified decision pathway, the model reinforces pathophysiological reasoning and highlights the complementary nature of these diagnostic domains.

The present study describes the development and proof-of-concept application of this integrated diagnostic flow model using fully synthetic emergency cases. The framework is intended for medical student education and serves as a reproducible foundation for future learner-based studies evaluating usability, perceived realism, and perceived educational value, including its impact on diagnostic reasoning performance. In the main text, we illustrate the framework using a representative pathology—acute mesenteric ischemia—and provide representative anonymized imaging examples to demonstrate how radiologic patterns populate the structured diagnostic nodes.

## 2. Materials and Methods

### 2.1. Study Design

This study was designed as a simulation-based educational research project aimed at developing and demonstrating an integrated diagnostic framework that unifies radiological imaging findings and biochemical laboratory data for emergency clinical decision support. Because the study utilized fully synthetic cases created specifically for educational and methodological purposes, no real patient information, imaging data, or laboratory results were used; therefore, the project did not require ethical committee approval. The design focused on constructing visually intuitive diagnostic flow algorithms to illustrate how multimodal diagnostic elements converge during acute clinical evaluation.

The study consisted of three sequential phases:

(1) Framework Development:

An interdisciplinary team of radiology and biochemistry experts collaborated to identify key emergency conditions in which laboratory markers and imaging findings play complementary diagnostic roles. Pathophysiological mechanisms, typical radiological features, and relevant biochemical abnormalities were reviewed and synthesized to construct a unified decision-making architecture. This process resulted in the creation of an Integrated Radiology–Biochemistry Diagnostic Flow Framework, intended as a structured visual guide for learners.

(2) Simulation Case Construction:

Synthetic emergency scenarios were developed to represent realistic clinical presentations while ensuring complete anonymity and absence of patient-derived data. For each selected pathology, typical laboratory profiles and imaging findings were generated based on well-established diagnostic patterns reported in the literature. Each scenario was embedded into the diagnostic flow framework to demonstrate the sequential integration of multimodal findings.

(3) Proof-of-Concept Demonstration:

A representative diagnostic flow algorithm—acute mesenteric ischemia—was selected for detailed presentation within the main manuscript to exemplify the structure and functionality of the model. Additional emergency conditions, including complicated ileus, small-bowel perforation, acute pancreatitis, diverticulitis, pulmonary embolism, and intracranial hemorrhage, were developed using the same methodological principles and provided in the Supplementary Material.

This study design allowed for the creation of a comprehensive, interdisciplinary, and ethically exempt simulation model intended to enhance diagnostic reasoning skills among medical students and early trainees in emergency medicine.

### 2.2. Development of Synthetic Emergency Cases

Synthetic emergency cases were developed to provide realistic yet ethically exempt clinical scenarios that reflect the diagnostic complexity of acute conditions encountered in emergency medicine. The objective of this phase was to generate standardized case profiles that accurately illustrate the interplay between radiological manifestations and biochemical abnormalities without referencing or reproducing real patient data.

The development process followed a structured, multi-step approach:

(1) Selection of Emergency Conditions:

Pathologies were chosen based on their clinical importance, diagnostic urgency, and reliance on the combined interpretation of imaging and laboratory findings. Conditions included acute mesenteric ischemia, complicated ileus, small-bowel perforation, acute pancreatitis, diverticulitis, pulmonary embolism, and intracranial hemorrhage. These entities were selected because they require rapid, multimodal diagnostic integration and represent common high-stakes presentations in emergency practice.

(2) Definition of Key Radiological Features:

For each condition, characteristic imaging findings were identified from established radiological literature and expert consensus. These elements included bowel wall hypoenhancement and pneumatosis in mesenteric ischemia, free intraperitoneal air in perforation, peripancreatic fat stranding in pancreatitis, and hyperdense hemorrhage patterns in intracranial bleeding. Radiologic features were then translated into structured descriptors adaptable for placement within the diagnostic flow framework.

(3) Construction of Corresponding Biochemical Profiles:

Parallel to imaging features, representative laboratory findings were synthesized based on known pathophysiologic mechanisms. Examples include elevated serum lactate in ischemia, leukocytosis and CRP elevation in inflammatory conditions, hyperamylasemia and lipase elevation in pancreatitis, and D-dimer elevation in suspected pulmonary embolism. Numerical values and trends were modeled to reflect typical ranges encountered in clinical practice while avoiding any reproduction of real patient data.

(4) Integration of Clinical Context:

Each synthetic case was assigned a brief clinical vignette—including age group, presenting symptoms, and relevant clinical triggers—to simulate realistic emergency presentations. These vignettes were intentionally kept concise to emphasize the primacy of radiology–biochemistry integration while still providing essential contextual cues to guide diagnostic flow.

(5) Consistency and Educational Alignment:

All synthetic cases were reviewed for internal consistency, ensuring that laboratory abnormalities, imaging findings, and clinical cues aligned with established diagnostic patterns. The cases were then adapted to support a stepwise teaching structure, enabling learners to engage with the diagnostic process in a controlled, sequenced manner.

The resulting synthetic cases formed the backbone of the integrated diagnostic flow framework, serving as standardized educational tools for demonstrating how multimodal diagnostic data converge to support decision-making in emergency settings.

### 2.3. Construction of the Integrated Diagnostic Flow Framework

The development of the diagnostic flow framework followed a structured, three-tiered methodology designed to merge laboratory medicine and radiological imaging into a coherent decision-support tool. The process included (i) definition of core diagnostic nodes, (ii) mapping of inter-modality relationships, and (iii) visualization of step-wise algorithmic pathways tailored for emergency scenarios.

(i) Definition of Core Diagnostic Nodes

Each selected emergency condition was deconstructed into a set of core diagnostic nodes reflecting essential elements of clinical reasoning:

- Clinical trigger: the initial symptom or risk condition prompting further evaluation
- Laboratory assessment: key biochemical markers selected based on pathophysiology, temporal relevance, and diagnostic value
- Imaging indication and modality choice: decision point determining appropriate radiological study (e.g., US, non-contrast CT, contrast-enhanced CT angiography)
- Imaging findings: hallmark structural or functional imaging features associated with the condition
- Decision escalation/red flag: predefined criteria signaling need for urgent intervention or change in diagnostic pathway

These nodes ensure that the framework captures major branching points in diagnostic decision-making rather than simply presenting static results.

(ii) Mapping Inter-Modality Relationships

A key innovation of the framework was the explicit mapping of relationships between biochemical abnormalities and imaging features. For example, elevated serum lactate may trigger CTA in mesenteric ischemia; elevated D-dimer may trigger CT pulmonary angiography in suspected pulmonary embolism; marked lipase elevation may guide targeted pancreatic imaging in acute pancreatitis. The mapping was conducted via the following approach:

- For each laboratory marker, a threshold value or trend was defined (e.g., lactate > 2 mmol/L)
- For each imaging modality, key findings were identified and classified by their diagnostic weight (e.g., absence of bowel wall enhancement = high-risk)
- Arrows linking laboratory nodes to imaging nodes were drawn to visualize “if–then” logic (if lactate elevated → proceed to CTA/angiography)
- Red-flag nodes were connected to both laboratory and imaging nodes, indicating when emergent intervention is required

This mapping stage was grounded in the concept of integrative diagnostics, which emphasizes that diagnostic data from in vitro (laboratories) and in vivo (imaging) sources should not reside in silos but be aggregated to enhance accuracy and timeliness.

(iii) Visualization of Step-Wise Algorithmic Pathways

The final step was to transform the mapped relationships into a visually intuitive flow chart format. Each condition was represented as a diagnostic flow framework composed of:

- A start node (clinical trigger)
- Laboratory assessment branch
- Imaging indication branch
- Diagnostic conclusion node
- Escalation branch (red-flag/urgent management)

These flow diagrams were supplemented by tabular summaries listing:

- Key laboratory parameters (including threshold or trend)
- Imaging modality and typical findings
- “Decision triggers” for branching
- Educational notes for teaching purposes (e.g., “If lactate rising despite resuscitation → consider laparotomy”)

Visual design principles included clear color-coded pathways (e.g., green for normal path, orange for caution path, red for escalation), consistent symbol usage (diamonds for decision points, rectangles for actions), and minimal text to enhance cognitive load management.

#### 2.3.1. Alignment with Educational Objectives

The framework aligns with the educational aim of enhancing diagnostic reasoning among medical students by:

- Offering a scaffolded decision model that mirrors real-life emergency workflows
- Promoting pattern recognition across laboratory and imaging domains
- Reducing cognitive overload by presenting a systematic, integrated pathway rather than discrete data elements
- Facilitating teaching in small groups or self-directed learning, with visual and textual components complementing each other

#### 2.3.2. Pilot Use and Refinement

Although this article does not report a full effectiveness trial, the framework underwent preliminary review by a panel of radiology and laboratory medicine educators who assessed clarity, logical coherence, and educational suitability. Minor refinements were made to decision thresholds and branching logic based on feedback (for example, modifying biochemical cut-off values, clarifying imaging indication prompts). The flowcharts and tables have been standardized to ensure consistency across all included emergency scenarios.

#### 2.3.3. Radiology-Centered Modality Selection Rationale

Within the framework, modality selection is presented as a deliberate decision node aligned with real emergency workflows. CTA is prioritized in conditions where vascular patency, active bleeding, or perfusion assessment is the primary diagnostic target and rapid escalation is required (e.g., mesenteric ischemia, pulmonary embolism, aortic dissection). MRI is preferred when superior soft-tissue contrast is essential and subtle parenchymal or neuroaxial pathology must be characterized (e.g., epidural abscess, cauda equina syndrome), particularly when CT findings may be non-specific. Ultrasound (including Doppler) is highlighted as a first-line or bedside modality in scenarios where dynamic vascular flow assessment or radiation-free evaluation is advantageous (e.g., acute cholecystitis, ovarian/testicular torsion, DVT). This modality reasoning reinforces imaging-centered diagnostic logic while keeping laboratory markers as supportive escalation cues rather than primary diagnostic determinants

### 2.4. Intended Educational Use

The Integrated Radiology–Biochemistry Diagnostic Flow Framework was designed as a simulation-based educational tool to support structured multimodal diagnostic reasoning in emergency medicine. The standardized flow format enables reproducible case-based learning using fully synthetic scenarios without patient data. Potential applications include small-group workshops, self-directed learning modules, and interdisciplinary teaching sessions for medical students and early trainees.

### 2.5. Conceptual Validation and Refinement

This study was designed as a methodological proof-of-concept based entirely on fully synthetic emergency scenarios. Because no real patient data, human participants, or learner testing were involved, the framework was not evaluated through outcome-based educational measures. Instead, the development process focused on conceptual validation, aiming to ensure that each scenario and diagnostic pathway was clinically plausible, logically consistent, and presented in a clear and standardized format.

The framework was constructed using common diagnostic steps shared across high-acuity emergency conditions. A targeted review of emergency radiology and laboratory–imaging correlation literature was used to identify recurring decision components. Based on this foundation, each scenario was structured using the same core elements: an initial clinical trigger, relevant laboratory markers when applicable, an imaging indication, key imaging findings, and an explicit decision/escalation point. The draft algorithms were then revised through iterative refinement cycles to simplify overly complex branches, remove repetition, and unify terminology across different conditions.

Finally, the completed pathways were reviewed for clinical coherence and alignment with routine emergency decision-making. This content validation was performed through expert review involving radiology and biochemistry specialists, with particular attention to the appropriateness of laboratory thresholds, the suitability of imaging recommendations, fidelity of imaging findings to established patterns, and consistency of escalation logic across scenarios. Minor structural and layout edits were introduced to improve readability and allow the diagrams to be followed without additional explanation. No educational outcomes were collected at any stage; therefore, the framework is presented as a conceptually validated educational model intended for future empirical evaluation.

### 2.6. Synthetic Cases for Demonstrating the Diagnostic Framework

To demonstrate the functionality of the Integrated Radiology–Biochemistry Diagnostic Flow Framework, a set of fully synthetic emergency scenarios was generated. These cases were designed to mimic realistic clinical presentations while containing no identifiable patient data, ensuring that no ethical approval was required. Each scenario included a coherent combination of (i) clinical triggers, (ii) key laboratory markers, and (iii) characteristic imaging findings.

**Figure 1:**
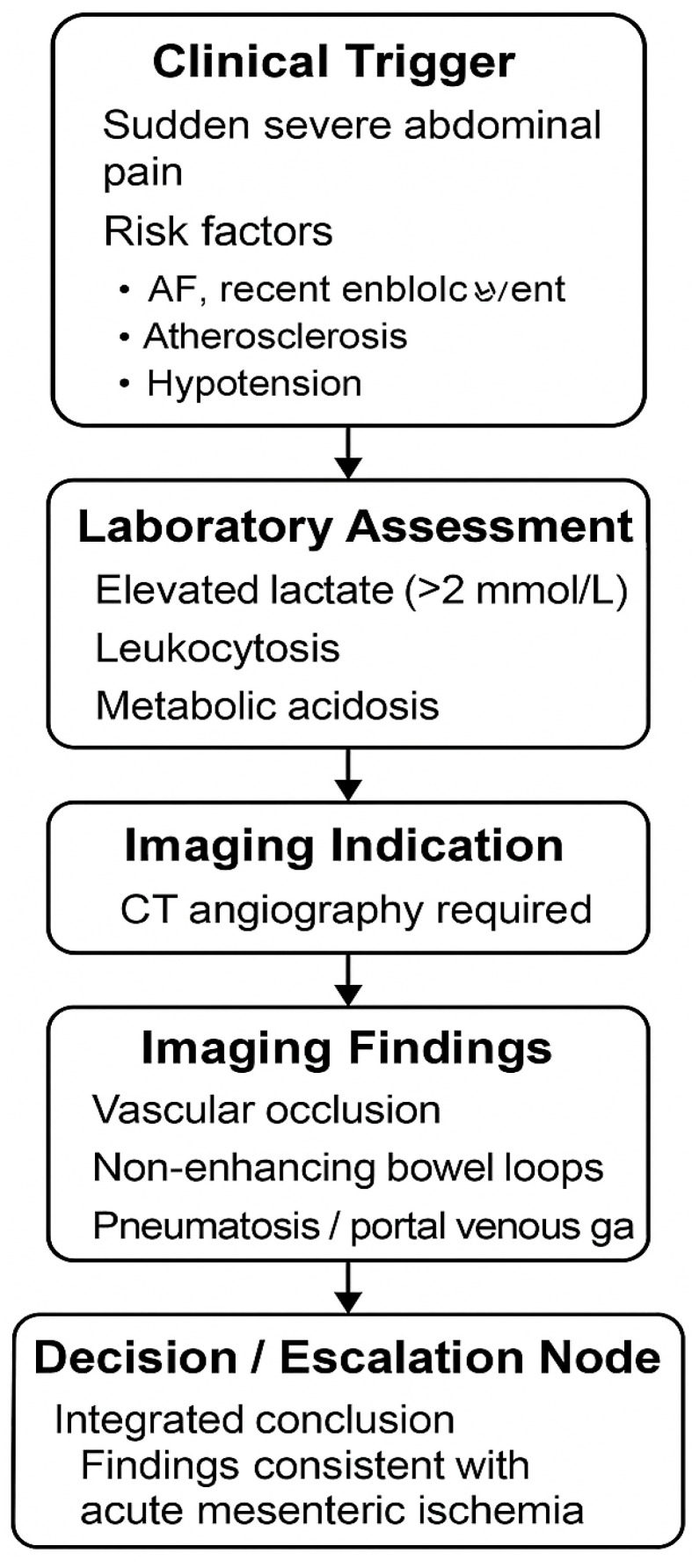
Structured radiology–biochemistry diagnostic flow illustrating integrated emergency decision-making in acute mesenteric ischemia.

A representative example—acute mesenteric ischemia—was selected for inclusion in the main manuscript to illustrate the complete flow from clinical presentation to the integrated diagnostic conclusion. The operational logic of this example is summarized visually in Figure 1, which depicts the sequential flow of clinical triggers, laboratory assessment, imaging indication, imaging findings, and escalation/decision nodes. The corresponding structured summary of this example is provided in Table 1: Representative Integrated Diagnostic Flow Framework Example: Acute Mesenteric Ischemia.

To demonstrate the breadth of applicability of the framework, 40 additional synthetic emergency conditions were constructed across abdominal, thoracic, neurological, vascular, trauma-related, and genitourinary domains. These cases were not meant to represent the full diagnostic complexity of real-world presentations but instead served as standardized inputs to test the coherence and internal consistency of the integrated approach. A consolidated overview of all 40 emergency conditions—including their key radiological and associated biochemical findings—is presented in Table 2: Overview of Emergency Radiology–Biochemistry Cases Included in the Study.

The complete set of fully expanded diagnostic pathways for these scenarios (Tables S1–S40), including stepwise clinical–laboratory–imaging integration, is provided in the Supplementary Materials.

### 2.7. Descriptive Framework Metrics

Because this study was a methodological proof-of-concept based entirely on fully synthetic emergency scenarios, no inferential statistical analyses or hypothesis testing were performed. The reported values represent descriptive framework metrics intended to document structural completeness and internal consistency of the Integrated Radiology–Biochemistry Diagnostic Flow Framework when applied to the synthetic case set. Specifically, coverage was summarized using counts and percentages for predefined mandatory domains (e.g., clinical trigger, risk factors, relevant laboratory markers when applicable, imaging findings, and explicit decision/escalation nodes). These metrics reflect design-level compliance of the framework rather than learner performance or clinical effectiveness.

### 2.8. Ethical Considerations

This study did not involve real patient data, imaging examinations, laboratory results, or any identifiable clinical information. All scenarios used in the development and evaluation of the Integrated Radiology–Biochemistry Diagnostic Flow Framework were fully synthetic and created solely for methodological demonstration. As no human participants, clinical interventions, or retrospective data extractions were included, approval from an institutional review board (IRB) or ethics committee was not required. The study adhered to the ethical principles outlined in the Declaration of Helsinki, although its scope did not involve human or animal research.

## 3. Results

This Results section reports framework-level outputs and descriptive internal consistency metrics obtained from the synthetic scenario set, rather than learner-based educational outcomes or clinical performance results.

### 3.1. Characteristics of the Synthetic Case Set

Because this study was based entirely on synthetic scenarios rather than real patient data, traditional demographic variables (age, sex, comorbidities) were not applicable. Instead, the synthetic cases were constructed to reflect a broad spectrum of emergency radiology indications commonly encountered in clinical practice. The final dataset included 40 distinct emergency conditions, each paired with characteristic laboratory markers and imaging findings.

For descriptive purposes, the scenarios were grouped into the four major domains of acute care radiology (Table 3):

- Neurological emergencies (*n* = 12; 30%): examples include intracerebral hemorrhage, ischemic stroke, subarachnoid hemorrhage, epidural hematoma, and cauda equina syndrome.
- Thoracic emergencies (*n* = 10; 25%): examples include pulmonary embolism, pneumothorax, pneumonia, pneumomediastinum, and foreign-body aspiration.
- Abdominal and pelvic emergencies (*n* = 14; 35%): examples include appendicitis, bowel obstruction, diverticulitis, pancreatitis, mesenteric ischemia, perforated viscus, cholecystitis, and ectopic pregnancy.
- Vascular and other acute pathologies (*n* = 4; 10%): examples include aortic dissection, ruptured abdominal aortic aneurysm, deep vein thrombosis, and orbital cellulitis.

### 3.2. Completeness of Mandatory Reporting Fields

Across the 40 synthetic emergency cases, completeness of the predefined mandatory domains varied according to the intrinsic clinical characteristics of each scenario. Clinical triggers, risk factors, imaging findings, and critical decision nodes were present in all cases (100%), reflecting their universal role in emergency diagnostic workflows. Laboratory/biochemistry data were included in 34 of 40 cases (85%), as six trauma-dominated scenarios (e.g., pneumothorax, testicular torsion, spinal trauma) did not require laboratory markers for diagnostic confirmation within the constructed templates.

The resulting completeness rates are summarized in Table 4.

### 3.3. Framework Application and Internal Consistency Metrics

The Integrated Radiology–Biochemistry Diagnostic Flow Framework was applied to 40 fully synthetic emergency scenarios to examine its internal consistency and structural completeness under simulation. Since all scenarios were intentionally standardized in format (clinical trigger, risk factors, laboratory markers when applicable, imaging findings, and an explicit decision/escalation node), the assessment focused on documenting whether the framework maintained a consistent stepwise structure.

Across the case set, heterogeneous inputs were organized into a unified, stepwise diagnostic pathway. In all 40 scenarios, the framework converged to a single predefined endpoint (e.g., surgical emergency, anticoagulation initiation, neurosurgical consultation, or conservative management) by design, reflecting framework-level convergence within the designed logic rather than clinical validation or learner performance.

Laboratory/biochemistry markers were incorporated in 34 of 40 scenarios (85%) in which they were defined as relevant within the synthetic templates. In these scenarios, laboratory variables were positioned within the escalation pathway to support coherence between biochemical and radiological reasoning (e.g., lactate in mesenteric ischemia, D-dimer in pulmonary embolism, lipase in acute pancreatitis). In the remaining six scenarios (15%) where laboratory markers were not applicable (e.g., imaging-driven traumatic pneumothorax, spinal trauma, testicular torsion), the framework operated through imaging-based decision routes without laboratory integration.

The framework promoted clarity by explicitly linking imaging and laboratory findings to management-oriented decision nodes. Common structural features included:

- Explicit categorization of critical findings to ensure visibility of life-threatening features.
- Standardized terminology across cases to reduce variability in expression for comparable findings.
- Consistent escalation cues directing high-risk patterns toward predefined endpoints.

A summary of internal consistency and structural completeness metrics is provided in Table 5.

**Figure 2:**
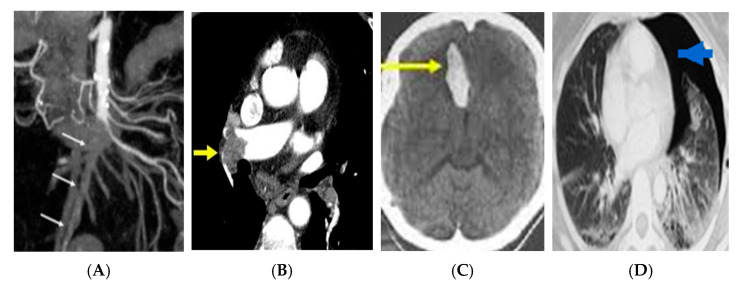
Representative anonymized imaging examples illustrating framework mapping across emergency scenarios. (**A**) Contrast-enhanced abdominal CT demonstrating imaging features consistent with acute mesenteric ischemia (mesenteric vessel occlusion; arrows). (**B**) CT pulmonary angiography demonstrating a pulmonary arterial filling defect consistent with acute pulmonary embolism (arrow). (**C**) Non-contrast head CT demonstrating acute intracranial hemorrhage (arrow). (**D**) Chest CT demonstrating pneumothorax (arrow).

Representative anonymized imaging examples illustrating how key radiologic patterns are mapped into the framework are provided in Figure 2.

These anonymized examples illustrate how key imaging findings populate the “Imaging Findings” domain and connect to explicit decision/escalation nodes within the framework.

### 3.4. Framework-Level Features Observed in the Synthetic Case Set

Across the forty fully synthetic emergency scenarios, the Integrated Radiology–Biochemistry Diagnostic Flow Framework generated standardized flowchart outputs that followed a consistent sequence from clinical trigger to laboratory consideration (when applicable), imaging selection, key radiologic pattern identification, and an explicit decision/escalation node. These outputs illustrate how the framework operationalizes multimodal diagnostic reasoning into a structured, radiology-centered pathway and how the same logic architecture can be applied across diverse emergency categories.

A notable framework feature was the explicit linkage of selected biochemical escalation cues to imaging-driven decision points in conditions where physiological markers are commonly used to guide urgency or diagnostic suspicion. For example, predefined thresholds or trajectories (e.g., lactate elevation in suspected mesenteric ischemia, D-dimer escalation in thromboembolic suspicion, or β-hCG context in early pregnancy emergencies) were integrated into branching logic to prompt modality escalation or refine differential prioritization. Conversely, scenarios dominated by imaging findings progressed through the framework without mandatory laboratory pathways, reflecting the imaging-anchored structure of the model.

The structured format also enabled clear visualization of red-flag routes leading to management-oriented endpoints. By requiring decisional closure for each scenario and organizing imaging findings within escalation branches, the framework outputs provide an interpretable reasoning trace that can be used in simulation-based teaching sessions or structured case discussions. However, these framework-level features represent implementation characteristics within a controlled synthetic dataset and should not be interpreted as evidence of clinical benefit, diagnostic performance improvement, or educational effectiveness.

### 3.5. Structural Consistency Across Emergency Domains

Within the limits of a simulation-based study, the Integrated Radiology–Biochemistry Diagnostic Flow Framework demonstrated structural robustness when applied to 40 synthetic emergency scenarios spanning neurological, thoracic, abdominal/pelvic, and vascular/other acute conditions. Because the evaluation was performed exclusively on predefined simulated cases—and not in a clinical environment—the term “robustness” refers to the internal consistency and uninterrupted function of the framework rather than real-world clinical performance.

Across all emergency categories, each case advanced through the sequential structure without generating contradictory branches, logical dead-ends, or incomplete decision pathways. In neurological and trauma-dominant scenarios, the framework progressed primarily through imaging-driven logic; in abdominal and vascular emergencies, laboratory markers—when present—were incorporated cohesively into the decision sequence. This demonstrated that the framework accommodated both imaging-only and multimodal (clinical + laboratory + imaging) patterns without structural instability.

These results indicate that the framework is structurally adaptable across diverse emergency case types within simulated conditions, supporting its utility as a controlled educational tool rather than a measure of clinical effectiveness.

### 3.6. Summary of Framework Outputs and Intended Use

Taken together, the forty fully synthetic emergency scenarios and the Integrated Radiology–Biochemistry Diagnostic Flow Framework generated a consistent set of radiology-centered diagnostic flowchart outputs across diverse emergency presentations. For each scenario, the framework documented a structured reasoning sequence linking an initial clinical trigger to targeted laboratory consideration (when applicable), imaging modality selection, key radiologic pattern identification, and an explicit escalation or decision endpoint. These outputs provide a transparent and reproducible representation of multimodal diagnostic logic that can be used for simulation-based teaching sessions, structured case discussion, and framework demonstration purposes.

Importantly, the present results should be interpreted as framework-level implementation outputs derived from internally generated synthetic cases. The documented pathways reflect internal logical coherence and design consistency within the constructed vignettes, rather than validated evidence of improved diagnostic accuracy, clinical performance, or educational effectiveness. Future expert- and learner-based studies are required to evaluate usability, perceived realism, cognitive workload, and potential educational value in authentic training environments.

### 3.7. Representative Case Output: Acute Mesenteric Ischemia

A representative example illustrating how the Integrated Radiology–Biochemistry Diagnostic Flow Framework operates is provided in Table 1 using a synthetic case of acute mesenteric ischemia. This example demonstrates how clinical triggers, selected biochemical markers, and imaging findings are organized into a stepwise pathway that concludes with a predefined management-oriented endpoint within the simulated logic structure.

The scenario begins with a clinical presentation of severe abdominal pain and risk factors consistent with vascular compromise. Laboratory abnormalities—specifically elevated lactate—serve as an escalation cue that increases suspicion of ischemia within the framework and supports prioritization of urgent imaging. CT angiography findings indicating non-enhancing bowel segments represent the key discriminating radiologic pattern, directing the pathway toward an escalation node consistent with urgent surgical evaluation.

Overall, this representative scenario illustrates the intended application of the framework under controlled simulation conditions by making the reasoning pathway explicit and traceable from trigger to endpoint. This example is provided as a demonstrative template, and the remaining 39 synthetic emergency scenarios are presented in the Supplementary Material.

## 4. Discussion

This simulation-based methodological study developed an Integrated Radiology–Biochemistry Diagnostic Flow Framework to support emergency diagnostic reasoning through a structured, multimodal approach [11,12]. By combining clinical triggers, relevant laboratory markers (when applicable), imaging findings, and explicit decision nodes, the framework addresses a recognized limitation of acute care reporting: the absence of a unified structure that consistently links these elements to a clear management-oriented conclusion [13,14]. Unlike narrative radiology reports, which may vary in style and completeness, the proposed framework provides a standardized pathway aligned with emergency decision-making across diverse presentations, including neurological, thoracic, abdominal/pelvic, and vascular scenarios [15,16]. In this model, imaging findings constitute the primary diagnostic anchor that drives escalation and management-oriented conclusions, while laboratory markers provide supportive physiological context in selected conditions. The integration of laboratory data offered added interpretive support in conditions where biochemical markers influence urgency or diagnostic suspicion—such as lactate in mesenteric ischemia, D-dimer in pulmonary embolism, or β-hCG in ectopic pregnancy—while imaging-dominant cases progressed through the framework without reliance on laboratory values [17,18].

Importantly, these observations arise from a controlled simulation setting and should not be interpreted as evidence of clinical effectiveness; empirical evaluation in real learners and clinical workflows is required. Representative anonymized imaging examples illustrating how key radiologic patterns populate the diagnostic nodes are provided in Figure 2.

### 4.1. Interpretation of Findings

Overall, the framework outputs of this simulation-based study indicate that the Integrated Radiology–Biochemistry Diagnostic Flow Framework provides a coherent structure for synthesizing clinical context, relevant laboratory markers, and imaging findings in emergency scenarios. Although the dataset was intentionally standardized, the framework could accommodate both imaging-driven pathways and laboratory-supported escalation logic, which is particularly relevant in time-critical conditions where urgency emerges from combined physiological and anatomical information (e.g., ischemic bowel disease, ruptured ectopic pregnancy, and acute pulmonary embolism) [19,20]. The framework also promotes structured completeness by requiring an explicit management-oriented conclusion; however, because the vignettes were synthetic by design, these observations should be interpreted as conceptual feasibility and internal logical coherence rather than clinical efficacy.

### 4.2. Educational Implications

The present study is best interpreted as an early-stage educational framework development project rather than an evaluation of educational effectiveness. In emergency care, clinicians must rapidly integrate heterogeneous diagnostic inputs—clinical presentation, laboratory markers, and imaging findings—and translate them into time-sensitive, management-oriented decisions. However, medical training frequently introduces these domains in parallel rather than through a single structured reasoning sequence, requiring learners to perform interdisciplinary integration implicitly and inconsistently.

Within this context, the Integrated Radiology–Biochemistry Diagnostic Flow Framework is proposed as a simulation-based model that externalizes and structures multidisciplinary diagnostic reasoning. By guiding users from an initial clinical trigger to targeted laboratory consideration (when applicable), imaging selection, interpretation of key radiologic patterns, and an explicit escalation/decision node, the framework may support teaching sessions by making diagnostic steps visible, auditable, and open to feedback. Importantly, the framework remains imaging-centered, reflecting the pivotal role of imaging findings as the primary diagnostic anchor in many emergency scenarios, while biochemical markers are incorporated selectively as escalation cues in conditions where physiology substantially influences urgency or diagnostic suspicion.

From an educational perspective, the standardized format may facilitate structured case discussion by clarifying where reasoning pathways diverge, where escalation logic is incomplete, or where decisional closure is missing. Instructors may also use the stepwise flow structure to highlight how biochemical abnormalities modify interpretation and urgency in high-stakes conditions where anatomical and physiological signals interact, such as ischemic bowel disease, ruptured ectopic pregnancy, and acute pulmonary embolism [21,22]. Because the scenarios are fully synthetic and contain no patient identifiers, the framework and its scenario library can be shared and adapted across educational settings without ethical or regulatory constraints.

Nevertheless, the educational utility of the framework cannot be assumed without empirical validation. Framework development in simulation-based education commonly proceeds through sequential stages, beginning with content and face validity evaluation before learner-based trials. Accordingly, a logical next step would be expert-driven validation involving a small multidisciplinary panel (e.g., 3–5 radiology and emergency medicine experts) to assess clinical plausibility, clarity, realism, and usefulness. Such assessment could incorporate concordance measures and structured rating instruments, potentially including validated tools (e.g., usability questionnaires such as the System Usability Scale) adapted for educational frameworks. Following expert validation, learner-centered studies are required to evaluate usability, perceived realism, cognitive workload, and potential impact on diagnostic reasoning performance within authentic training environments.

### 4.3. Comparison with Previous Literature

Existing literature in emergency radiology has predominantly focused on improving diagnostic accuracy, optimizing imaging protocols, and enhancing communication through structured reporting formats tailored to specific clinical entities or anatomical regions [23,24]. Professional societies and expert panels have proposed reporting checklists and standardized elements for high-stakes scenarios such as trauma, acute stroke, pulmonary embolism, and abdominal emergencies, reflecting broad efforts to reduce ambiguity and variability in time-critical settings [25,26]. However, most structured reporting approaches remain imaging-centered and generally do not incorporate laboratory data or clinical triggers into the reporting logic.

In parallel, laboratory medicine research has established the diagnostic and triage value of numerous biochemical markers used in emergency care, including lactate in mesenteric ischemia, D-dimer in venous thromboembolism, β-hCG in early pregnancy assessment, and inflammatory indices in intra-abdominal infection [27,28]. Yet these contributions are typically presented within laboratory interpretation frameworks and rarely translated into explicit imaging-directed diagnostic pathways. As a result, the integration of laboratory escalation cues with radiologic decision-making is often left implicit, depending on individual clinician experience and local workflow.

A limited body of work has addressed multidisciplinary diagnostic reasoning, including simulation-based education and integrated case discussions; however, such integration is commonly presented at a conceptual level rather than as an operational, stepwise algorithm applicable across diverse emergency presentations [29,30]. In this context, the present study differs in its methodological focus: it proposes an explicit diagnostic flow framework that organizes clinical triggers, selected biochemical parameters, imaging modality selection, key radiologic patterns, and escalation decision nodes within a unified structure.

Importantly, the framework should be interpreted as a simulation-based proof-of-concept rather than an effectiveness study. It is not intended to replace disease-specific structured reporting templates or clinical guidelines, but rather to complement existing educational strategies by making interdisciplinary diagnostic reasoning more visible and systematically teachable. By documenting internal logic coherence across forty synthetic emergency scenarios, this study provides a reproducible foundation for future learner- and expert-based validation research in emergency diagnostic education.

### 4.4. Novel Aspects and Added Value of This Study

This study offers a simulation-based methodological contribution by proposing a structured diagnostic flow framework that integrates radiology-centered imaging interpretation with selected biochemical escalation cues in emergency presentations. Rather than functioning as a clinically validated decision-support system, the framework is intended as an educational and conceptual model that makes multidisciplinary diagnostic reasoning explicit, traceable, and reproducible within controlled learning environments.

A key added value of the proposed model is its emphasis on stepwise integration. In many emergency scenarios, diagnostic reasoning progresses dynamically from an initial clinical trigger to targeted laboratory evaluation (when applicable), followed by imaging selection and interpretation, and finally to a management-oriented conclusion. The present framework operationalizes this reasoning sequence in a standardized flow structure, allowing learners to visualize how imaging findings act as the primary diagnostic anchor while biochemical markers may modify diagnostic probability or urgency in selected conditions.

Another contribution of this study lies in the use of explicit decision nodes and escalation pathways. Instead of presenting radiologic findings as isolated descriptive outputs, the model organizes critical imaging patterns within branching logic that highlights red-flag routes requiring urgent intervention. This design supports structured completeness and may reduce variability in case interpretation during simulation-based teaching sessions by clarifying missing steps, inappropriate escalation logic, or incomplete decisional closure.

In addition, the creation of a fully synthetic scenario library provides a reproducible resource for standardized training and evaluation. Synthetic vignettes enable controlled case generation across diverse emergency categories, avoid ethical and regulatory barriers associated with patient-derived datasets, and allow consistent implementation of the framework without privacy concerns. Importantly, the scenario set in this manuscript is not presented as evidence of real-world performance but as a tool demonstrating internal logical coherence and framework applicability across representative emergency conditions.

Overall, the framework’s main contribution is to provide an operational structure for interdisciplinary diagnostic reasoning that can serve as a foundation for future research. Subsequent work should incorporate expert-based content validation and learner-centered testing to assess realism, usability, and potential educational effectiveness before any clinical translation is considered.

### 4.5. Strengths and Limitations

This study has several strengths. Most notably, it proposes a unified diagnostic framework that explicitly integrates clinical triggers, relevant laboratory markers (when applicable), imaging findings, and decision/escalation nodes within a single structured pathway. The exclusive use of fully synthetic scenarios enabled the framework to be developed and applied without ethical constraints and allowed balanced representation across major emergency categories. In addition, the standardized scenario format ensured conceptual consistency across conditions and permitted examination of the framework’s internal logic without confounding variability related to documentation style, institutional reporting habits, or workflow heterogeneity. Finally, the manuscript consistently frames the proposed model as a methodological proof-of-concept and avoids claims of clinical validation.

Several limitations should be clearly acknowledged. First, this proof-of-concept phase included no learners and no real educational implementation. Specifically: (i) no learners were enrolled; (ii) no comparison group was included; (iii) no expert panel review or concordance assessment was conducted; (iv) no usability testing was performed; and (v) no educational outcomes (e.g., diagnostic reasoning performance, learning gain, or skill acquisition metrics) were measured. Therefore, external validity, user acceptability, and educational effectiveness cannot be inferred from the present work. In addition, all cases were fully synthetic and internally generated, meaning that findings reflect framework-level behavior under standardized simulated conditions rather than real-world clinical complexity.

The study was conducted entirely in a controlled simulation setting and was not implemented within real clinical workflows. Accordingly, no real-image viewing sessions on clinical workstations (e.g., PACS environments) were performed as part of this proof-of-concept design. Because scenarios were intentionally constructed with predefined diagnostic elements, completeness metrics reflect template characteristics rather than actual reporting behavior. Moreover, synthetic vignettes may not capture the full heterogeneity, ambiguity, and evolving context of real emergency presentations. No reader study or interobserver comparison was performed; therefore, the impact of the framework on reporting consistency, diagnostic accuracy, clinical communication, or decision-making under uncertainty remains unknown. In addition, the framework was not evaluated within PACS/RIS environments, and practical issues related to workflow integration and user experience require further investigation.

Taken together, these strengths and limitations support interpretation of the present study as an educational and methodological proof-of-concept, providing a structured foundation for future expert-based and learner-based validation studies.

### 4.6. Future Directions

Future work should focus on advancing the Integrated Radiology–Biochemistry Diagnostic Flow Framework beyond the current controlled simulation-based proof-of-concept and toward empirical validation in environments that more closely approximate real educational and clinical workflows. As an initial validation step prior to learner-based implementation, an expert-driven evaluation is warranted. This may include a small multidisciplinary panel (e.g., 3–5 radiology and emergency medicine experts) to assess clinical plausibility, clarity, usefulness, and realism of both the framework structure and the synthetic scenarios. Expert review could be complemented by concordance analysis to quantify agreement regarding key decision nodes, escalation logic, and management-oriented endpoints. Where feasible, structured scoring instruments should be used, including validated usability tools such as the System Usability Scale (SUS) and other educational usability or acceptability measures.

Following expert validation, the next step would be a learner-centered reader study involving radiology trainees, emergency medicine residents, or multidisciplinary learners to evaluate usability, reasoning traceability, reporting completeness, and communication clarity compared with unstructured narrative approaches. Such studies may also assess perceived realism, cognitive workload, and learner satisfaction, and help identify components requiring refinement or interface optimization.

Another promising direction is incorporation of the framework into digital educational platforms, such as interactive modules or simulation software, enabling decision tracing, automated feedback, and repeated practice across progressively complex cases. Embedding the model into PACS-like training environments may further clarify practical issues related to workflow integration, user experience, and interface design.

Expanding the synthetic case library also represents an important opportunity. While the current set captures major emergency categories, future scenario sets could incorporate multimorbidity, equivocal imaging findings, incomplete laboratory profiles, or evolving trajectories to better reflect real-world diagnostic uncertainty. Such expansion would support tiered curricula tailored to different training levels and may facilitate benchmarking of reasoning processes.

Finally, once expert-based feasibility and learner acceptability are established, subsequent research could explore clinical-facing validation questions, including whether the framework may support interobserver consistency, structured completeness, and interdisciplinary communication. Such work would be essential before considering any translation into routine clinical practice.

## 5. Conclusions

This simulation-based methodological study introduces an Integrated Radiology–Biochemistry Diagnostic Flow Framework that unifies clinical triggers, laboratory markers, imaging findings, and explicit decision nodes into a single structured pathway for emergency scenarios. Applied to 40 synthetic cases, the framework demonstrated internal consistency, decisional clarity, and adaptability across diverse acute presentations, highlighting its potential value as an educational tool for teaching multimodal diagnostic reasoning.

While the findings reflect structural performance within controlled synthetic conditions rather than clinical effectiveness, the model provides a reproducible foundation for strengthening diagnostic integration in training environments. By promoting completeness, standardization, and actionable interpretation, the framework addresses a recognized need for clearer and more coherent decision pathways in emergency-focused education.

Future research should evaluate the model in real-world training settings, incorporate user feedback, and explore its integration into digital simulation platforms. With further refinement and empirical validation, the framework may contribute to improved interdisciplinary understanding and more consistent diagnostic approaches in acute care education. Future studies are warranted to validate this proof-of-concept framework through learner participation, usability testing, and assessment of educational outcomes.

## Figures and Tables

**Table 1:** Representative Integrated Diagnostic Flow Framework Example: Acute Mesenteric Ischemia.

Diagnostic Component	Node/Criteria	Description/Action
1. Clinical Trigger	Sudden severe abdominal pain	Disproportionate to physical exam; consider high-risk presentation.
	Risk factors	Atrial fibrillation, recent embolic event, atherosclerosis, hypotension.
2. Laboratory Assessment	Elevated lactate (>2 mmol/L)	Indicates tissue hypoperfusion; proceed to urgent imaging.
	Leukocytosis	Supportive but non-specific inflammatory indicator.
	Metabolic acidosis	Suggests advanced ischemia or systemic hypoperfusion.
3. Imaging Indication	CT angiography required	CTA is recommended when clinical and laboratory criteria converge.
4. Imaging Findings	Vascular occlusion	Superior mesenteric artery thrombus/embolus.
	Non-enhancing bowel loops	Strong radiologic indicator of ischemia or infarction.
	Pneumatosis intestinalis or portal venous gas	Suggests irreversible ischemia; requires immediate escalation.
5. Decision/Escalation Node	Integrated conclusion	“Findings consistent with acute mesenteric ischemia.”
	Recommended action	Immediate surgical consultation ± endovascular intervention.

**Table 2:** Overview of Emergency Radiology–Biochemistry Cases Included in the Study.

Case No	Emergency Condition	Imaging Modality	Key Radiological Finding	Key Biochemistry Finding
1	Acute appendicitis	CT abdomen	Dilated appendix with wall thickening	Leukocytosis, ↑CRP
2	Bowel obstruction	CT abdomen	Dilated loops with air–fluid levels	Electrolyte imbalance (↓K^+^, ↓Cl^−^)
3	Intracerebral hemorrhage	Non-contrast CT	Hyperdense parenchymal hematoma	Normal labs or ↑BP-related parameters
4	Ischemic stroke	CT perfusion	Perfusion mismatch	Normal labs or mild ↑D-dimer
5	Subarachnoid hemorrhage	CT brain	Hyperdensity in basal cisterns	Possible ↑WBC (stress leukocytosis)
6	Pulmonary embolism	CTPA	Intraluminal filling defect	↑D-dimer (strong trigger node)
7	Aortic dissection	CTA	Intimal flap	↑D-dimer (supportive)
8	Ruptured AAA	CTA	Retroperitoneal hematoma	↓Hemoglobin, ↓Hematocrit
9	Traumatic hemothorax	CT thorax	Hyperdense pleural fluid	↓Hemoglobin
10	Traumatic pneumothorax	CT thorax	Pleural air with lung collapse	Typically normal labs
11	Splenic injury	CT abdomen	Parenchymal laceration	↓Hemoglobin
12	Liver injury	CT abdomen	Lacerations with free fluid	↑AST/ALT
13	Renal colic	Non-contrast CT	Ureteral stone	Microscopic hematuria
14	Pyelonephritis	Contrast CT	Striated nephrogram	↑WBC, ↑CRP
15	Acute cholecystitis	Ultrasound	Wall thickening, stones	↑WBC, ↑CRP
16	Gallstone ileus	CT abdomen	Ectopic gallstone	
17	Mesenteric ischemia	CTA	Non-enhancing loops, occlusion	↑Lactate, metabolic acidosis
18	Perforated viscus	CT abdomen	Free intraperitoneal air	↑WBC, ↑CRP
19	Pancreatitis	CT abdomen	Pancreatic edema, fat stranding	↑Amylase/Lipase
20	Diverticulitis	CT abdomen	Segmental wall thickening	↑WBC, ↑CRP
21	Ovarian torsion	Doppler US	Reduced vascularity	↑WBC (late)
22	Ectopic pregnancy	US	Adnexal mass with hemoperitoneum	↑β-hCG
23	Testicular torsion	Doppler US	Absent flow	Typically normal labs
24	Small bowel perforation	CT abdomen	Localized free air	↑WBC, ↑CRP
25	Peritonitis	CT abdomen	Diffuse peritoneal enhancement	↑WBC, ↑CRP, ↑Lactate
26	Pulmonary edema	CXR	Perihilar opacities	↑BNP
27	Pneumonia	CXR	Focal consolidation	↑WBC, ↑CRP
28	COVID-19 pneumonia	CT thorax	Peripheral ground-glass opacities	↓Lymphocytes, ↑CRP, ↑Ferritin
29	Pneumomediastinum	CT thorax	Free mediastinal air	Normal labs
30	MI complication	Cardiac CT	LV aneurysm	↑Troponin (acute phase)
31	DVT	Doppler US	Non-compressible vein	↑D-dimer
32	Stroke mimic	CT/MRI	No vascular occlusion	↑Lactate (post-ictal), ↑CK
33	Spinal trauma	CT spine	Vertebral fracture	↓Hemoglobin (if bleeding)
34	Cauda equina syndrome	MRI	Large disk herniation	Normal labs
35	Epidural abscess	MRI	Rim-enhancing collection	↑WBC, ↑CRP
36	Subdural hematoma	CT brain	Crescent-shaped collection	Normal labs
37	Epidural hematoma	CT brain	Biconvex collection	Normal labs
38	Cerebral venous thrombosis	MRV	Absent venous flow	↑D-dimer
39	Orbital cellulitis	CT orbit	Fat stranding, abscess	↑WBC, ↑CRP
40	Foreign body aspiration	CXR	Radiopaque object	Normal labs

Note: Complete integrated algorithms for all 40 cases are presented in Supplementary Tables S1–S40. Arrows indicate direction of laboratory change (↑ = increased, ↓ = decreased).

**Table 3:** Distribution of Synthetic Emergency Cases Across Clinical Domains.

Emergency Domain	*n*	%
Neurological emergencies	12	30%
Thoracic emergencies	10	25%
Abdominal/pelvic emergencies	14	35%
Vascular and other acute pathologies	4	10%
Total	40	100%

**Table 4:** Completeness of Mandatory Reporting Domains Across 40 Synthetic Cases.

Mandatory Domain	Present (n)	Missing (n)	Completeness (%)
Clinical Information	40	0	100%
Risk Factors	40	0	100%
Laboratory/Biochemistry Data	34	6	85%
Imaging Findings	40	0	100%
CriticalFindings/Decision Node	40	0	100%

**Table 5:** Summary of Framework Metrics (Internal Consistency and Coverage).

Framework Metric	Observation Across Synthetic Scenarios
Convergence to a predefined actionable endpoint	Observed in all 40 scenarios (40/40; 100%)
Inclusion of laboratory markers when applicable	Laboratory markers were included in 34 scenarios (34/40; 85%); not applicable in 6 imaging-driven scenarios (6/40; 15%)
Presence of an explicit decision/escalation node	Present in all scenarios (40/40; 100%)
Completeness of mandatory reporting domains (clinical trigger, imaging findings, endpoint)	Fully represented in all scenarios by template design (40/40; 100%)
Cross-domain applicability	Applicable across neurological, thoracic, abdominal/pelvic, and vascular emergency categories
Standardization of terminology and structured flow	Ensured by the framework template across all scenarios

## Data Availability

The synthetic datasets generated and analyzed during this study are available from the corresponding author on reasonable request.

## Supplementary Materials

The following supporting information can be downloaded at: https://www.mdpi.com/article/10.3390/tomography12020016/s1, Supplementary Materials provide the complete set of 40 synthetic emergency scenarios used in this study. Each scenario includes a structured application of the Integrated Radiology–Biochemistry Diagnostic Flow Framework, following the same multimodal format demonstrated for the representative case in the main text. The Supplementary Tables (S1–S40) present case-specific integrations of clinical triggers, risk factors, laboratory markers (when applicable), imaging findings, and final decision nodes. These materials are intended to illustrate the reproducibility of the framework across diverse emergency conditions and to support its use as a simulation-based educational tool.
