# Supplementary material for: Integrated Radiology–Biochemistry Diagnostic Flow Framework for Emergency Clinical Decision Support: A Simulation-Based Educational Model
Source: Tomography. 2026 Jan 27;12(2):16. doi: 10.3390/tomography12020016 (PMC12944950; doi:10.3390/tomography12020016)
Supplement: Supplementary file 1 [file tomography-12-00016-s001.zip › tomography-4127506-supplementary.pdf]

# SUPPLEMENTARY TABLES S1–S40

## Supplementary Table S1. Acute Appendicitis

| Component | Content |
|---|---|
| Clinical Trigger | RLQ pain, fever, nausea |
| Risk Factors | Young age, appendicolith |
| Key Biochemistry | Leukocytosis, ↑CRP |
| Imaging Findings | Dilated appendix, wall thickening, fat stranding |
| Decision / Escalation Node | Acute appendicitis — surgical consultation |

## Supplementary Table S2. Bowel Obstruction

| Component | Content |
|---|---|
| Clinical Trigger | Abdominal distension, vomiting, constipation |
| Risk Factors | Prior abdominal surgery, hernia |
| Key Biochemistry | Electrolyte imbalance (↓K, ↓Na), dehydration markers |
| Imaging Findings | Dilated loops, air–fluid levels, transition point |
| Decision / Escalation Node | Mechanical obstruction — surgical evaluation |

## Supplementary Table S3. Intracerebral Hemorrhage

| Component | Content |
|---|---|
| Clinical Trigger | Sudden headache, vomiting, focal deficit |
| Risk Factors | Hypertension, anticoagulant use |
| Key Biochemistry | INR elevation, platelet abnormalities |
| Imaging Findings | Hyperdense parenchymal hematoma |
| Decision / Escalation Node | Acute ICH — neurosurgical consultation |

## Supplementary Table S4. Ischemic Stroke

| Component | Content |
|---|---|
| Clinical Trigger | Acute focal neurological deficit |

| Component | Content |
| --- | --- |
| Risk Factors | AFib, carotid disease |
| Key Biochemistry | Glucose, coagulation panel |
| Imaging Findings | Perfusion mismatch, early ischemic changes |
| Decision / Escalation Node | Candidate for thrombolysis/thrombectomy (protocol-based) |

## Supplementary Table S5. Subarachnoid Hemorrhage

| Component | Content |
| --- | --- |
| Clinical Trigger | "Worst headache", neck stiffness |
| Risk Factors | Aneurysm, hypertension |
| Key Biochemistry | Coagulation markers (PT/INR) |
| Imaging Findings | Hyperdensity in basal cisterns and sulci |
| Decision / Escalation Node | SAH — urgent neurosurgical evaluation |

## Supplementary Table S6. Pulmonary Embolism

| Component | Content |
| --- | --- |
| Clinical Trigger | Dyspnea, chest pain, hypoxia |
| Risk Factors | Immobilization, cancer, DVT history |
| Key Biochemistry | ↑D-dimer, ABG abnormalities |
| Imaging Findings | Filling defect in pulmonary arteries |
| Decision / Escalation Node | Acute PE — initiate anticoagulation / PE response protocol |

## Supplementary Table S7. Aortic Dissection

| Component | Content |
| --- | --- |
| Clinical Trigger | Sudden tearing chest/back pain |

| Component | Content |
|---|---|
| Risk Factors | Hypertension, connective tissue disorders |
| Key Biochemistry | D-dimer elevation (supportive) |
| Imaging Findings | Intimal flap separating lumens |
| Decision / Escalation Node | Type A — emergent surgery; Type B — BP control |

## Supplementary Table S8. Ruptured Abdominal Aortic Aneurysm

| Component | Content |
|---|---|
| Clinical Trigger | Severe abdominal/back pain, hypotension |
| Risk Factors | Age > 65, smoking |
| Key Biochemistry | Hb drop, lactate elevation |
| Imaging Findings | Retroperitoneal hematoma adjacent to aorta |
| Decision / Escalation Node | Surgical emergency |

## Supplementary Table S9. Traumatic Hemothorax

| Component | Content |
|---|---|
| Clinical Trigger | Chest trauma, dyspnea |
| Risk Factors | High-energy trauma |
| Key Biochemistry | Hb drop |
| Imaging Findings | Hyperdense pleural collection |
| Decision / Escalation Node | Chest tube placement |

## Supplementary Table S10. Traumatic Pneumothorax

| Component | Content |
|---|---|
| Clinical Trigger | Chest trauma, shortness of breath |

| Component | Content |
| --- | --- |
| Risk Factors | Rib fractures |
| Key Biochemistry | N/A |
| Imaging Findings | Pleural air, lung collapse |
| Decision / Escalation Node | Needle decompression / chest tube |

## Supplementary Table S11. Splenic Injury

| Component | Content |
| --- | --- |
| Clinical Trigger | Abdominal trauma, LUQ pain |
| Risk Factors | High-energy trauma |
| Key Biochemistry | Hb drop |
| Imaging Findings | Laceration with perisplenic fluid |
| Decision / Escalation Node | Grading-based management (non-operative vs operative) |

## Supplementary Table S12. Liver Injury

| Component | Content |
| --- | --- |
| Clinical Trigger | RUQ trauma |
| Risk Factors | Seatbelt sign, high-speed collision |
| Key Biochemistry | Elevated AST/ALT |
| Imaging Findings | Hypodense lacerations, perihepatic fluid |
| Decision / Escalation Node | Non-operative management if stable |

## Supplementary Table S13. Renal Colic

| Component | Content |
| --- | --- |
| Clinical Trigger | Flank pain radiating to groin |

| Component | Content |
| --- | --- |
| Risk Factors | Prior stones, dehydration |
| Key Biochemistry | Hematuria, ↑creatinine (possible) |
| Imaging Findings | Hyperdense ureteral stone, proximal dilatation |
| Decision / Escalation Node | Analgesia, hydration, urology referral if large stone |

## Supplementary Table S14. Pyelonephritis

| Component | Content |
| --- | --- |
| Clinical Trigger | Fever, flank pain |
| Risk Factors | Diabetes, obstruction |
| Key Biochemistry | Leukocytosis, ↑CRP, abnormal urine test |
| Imaging Findings | Striated nephrogram, perinephric fat stranding |
| Decision / Escalation Node | IV antibiotics; admit if severe |

## Supplementary Table S15. Acute Cholecystitis

| Component | Content |
| --- | --- |
| Clinical Trigger | RUQ pain, fever |
| Risk Factors | Gallstones |
| Key Biochemistry | ↑WBC, ↑CRP, mild ↑LFTs |
| Imaging Findings | Distended gallbladder, wall thickening |
| Decision / Escalation Node | Cholecystectomy evaluation |

## Supplementary Table S16. Gallstone Ileus

| Component | Content |
| --- | --- |
| Clinical Trigger | Abdominal distension, vomiting |
| Risk Factors | Elderly, gallstones |

| Component | Content |
| --- | --- |
| Key Biochemistry | Electrolyte disturbances |
| Imaging Findings | Ectopic gallstone, bowel obstruction |
| Decision / Escalation Node | Surgical management |

## Supplementary Table S17. Mesenteric Ischemia

| Component | Content |
| --- | --- |
| Clinical Trigger | Severe disproportionate abdominal pain |
| Risk Factors | AFib, embolic risk |
| Key Biochemistry | ↑Lactate, leukocytosis |
| Imaging Findings | Non-enhancing loops, vessel occlusion |
| Decision / Escalation Node | Acute ischemia — surgical emergency |

## Supplementary Table S18. Perforated Hollow Viscus

| Component | Content |
| --- | --- |
| Clinical Trigger | Sudden abdominal pain, peritonitis |
| Risk Factors | PUD, NSAID use |
| Key Biochemistry | Leukocytosis |
| Imaging Findings | Free intraperitoneal air |
| Decision / Escalation Node | Emergent surgery |

## Supplementary Table S19. Pancreatitis

| Component | Content |
| --- | --- |
| Clinical Trigger | Epigastric pain radiating to back |
| Risk Factors | Gallstones, alcohol |
| Key Biochemistry | ↑Lipase, ↑Amylase |

| Component | Content |
|---|---|
| Imaging Findings | Enlarged pancreas, fat stranding |
| Decision / Escalation Node | Supportive management |

## Supplementary Table S20. Diverticulitis

| Component | Content |
|---|---|
| Clinical Trigger | LLQ pain, fever |
| Risk Factors | Age > 50 |
| Key Biochemistry | ↑WBC, ↑CRP |
| Imaging Findings | Segmental wall thickening, fat stranding |
| Decision / Escalation Node | Antibiotics; surgery if complicated |

## Supplementary Table S21. Ovarian Torsion — Integrated Diagnostic Flow

| Component | Content |
|---|---|
| Clinical Trigger | Sudden pelvic pain, nausea/vomiting |
| Risk Factors | Ovarian cyst, reproductive age |
| Key Biochemistry | Mild leukocytosis; β-hCG to exclude pregnancy |
| Imaging Findings | Enlarged ovary, absent/reduced Doppler flow |
| Decision / Escalation Node | GYN surgical emergency |

## Supplementary Table S22. Ectopic Pregnancy

| Component | Content |
|---|---|
| Clinical Trigger | Pelvic pain, vaginal bleeding |
| Risk Factors | Prior ectopic, IVF |
| Key Biochemistry | ↑β-hCG without intrauterine sac |

| Component | Content |
|---|---|
| Imaging Findings | Adnexal mass, hemoperitoneum |
| Decision / Escalation Node | Immediate gynecologic evaluation |

## Supplementary Table S23. Testicular Torsion

| Component | Content |
|---|---|
| Clinical Trigger | Sudden scrotal pain |
| Risk Factors | Bell-clapper deformity |
| Key Biochemistry | N/A |
| Imaging Findings | Enlarged hypoechoic testis, absent Doppler flow |
| Decision / Escalation Node | Urologic emergency — detorsion required |

## Supplementary Table S24. Small Bowel Perforation

| Component | Content |
|---|---|
| Clinical Trigger | Acute abdomen, peritoneal signs |
| Risk Factors | Ischemia, trauma, Crohn’s |
| Key Biochemistry | Leukocytosis, ↑CRP |
| Imaging Findings | Localized free air; adjacent fluid |
| Decision / Escalation Node | Emergency surgery |

## Supplementary Table S25. Peritonitis

| Component | Content |
|---|---|
| Clinical Trigger | Diffuse abdominal pain, guarding |
| Risk Factors | Perforation, PID, cirrhosis |
| Key Biochemistry | ↑WBC, ↑CRP, metabolic acidosis |
| Imaging Findings | Diffuse peritoneal enhancement with free fluid |

| Component | Content |
|---|---|
| Decision / Escalation Node | Immediate surgical/medical management |

## Supplementary Table S26. Pulmonary Edema

| Component | Content |
|---|---|
| Clinical Trigger | Dyspnea, orthopnea |
| Risk Factors | CHF, renal failure |
| Key Biochemistry | ↑BNP, ABG abnormality |
| Imaging Findings | Bilateral perihilar opacities, vascular redistribution |
| Decision / Escalation Node | Diuretics; treat underlying cause |

## Supplementary Table S27. Pneumonia

| Component | Content |
|---|---|
| Clinical Trigger | Fever, cough, dyspnea |
| Risk Factors | Elderly, COPD |
| Key Biochemistry | Leukocytosis, ↑CRP |
| Imaging Findings | Focal consolidation, air bronchogram |
| Decision / Escalation Node | Antibiotic therapy |

## Supplementary Table S28. COVID-19 Pneumonia

| Component | Content |
|---|---|
| Clinical Trigger | Fever, cough, hypoxia |
| Risk Factors | Exposure, chronic disease |
| Key Biochemistry | ↑CRP, ↑D-dimer, lymphopenia |
| Imaging Findings | Bilateral peripheral ground-glass opacities |
| Decision / Escalation Node | O2 therapy; COVID protocol |

## Supplementary Table S29. Pneumomediastinum

| Component | Content |
|---|---|
| Clinical Trigger | Chest pain, dyspnea, subcutaneous emphysema |
| Risk Factors | Asthma exacerbation, Valsalva |
| Key Biochemistry | N/A |
| Imaging Findings | Free mediastinal air outlining structures |
| Decision / Escalation Node | Conservative vs evaluate for underlying cause |

## Supplementary Table S30. Myocardial Infarction Complication (LV Aneurysm)

| Component | Content |
|---|---|
| Clinical Trigger | Chest pain, heart failure signs |
| Risk Factors | Prior MI |
| Key Biochemistry | ↑Troponin, ↑BNP |
| Imaging Findings | Ventricular wall thinning, aneurysm formation |
| Decision / Escalation Node | Cardiology evaluation |

## Supplementary Table S31. Deep Vein Thrombosis

| Component | Content |
|---|---|
| Clinical Trigger | Leg swelling, tenderness |
| Risk Factors | Immobilization, cancer |
| Key Biochemistry | ↑D-dimer |
| Imaging Findings | Non-compressible vein; intraluminal thrombus |
| Decision / Escalation Node | Anticoagulation |

## Supplementary Table S32. Stroke Mimic (Seizure-Related)

| Component | Content |
|---|---|
| Clinical Trigger | Transient neurological deficit |

| Component | Content |
| --- | --- |
| Risk Factors | Epilepsy |
| Key Biochemistry | Normal; lactate may be transiently ↑ |
| Imaging Findings | Cortical signal changes; no vessel occlusion |
| Decision / Escalation Node | Exclude stroke; neurologic evaluation |

## Supplementary Table S33. Spinal Trauma

| Component | Content |
| --- | --- |
| Clinical Trigger | Trauma with back pain, neurological deficit |
| Risk Factors | High-energy injury |
| Key Biochemistry | N/A |
| Imaging Findings | Vertebral fracture with retropulsion |
| Decision / Escalation Node | Spine surgery evaluation |

## Supplementary Table S34. Cauda Equina Syndrome

| Component | Content |
| --- | --- |
| Clinical Trigger | Saddle anesthesia, urinary retention |
| Risk Factors | Large disc herniation |
| Key Biochemistry | N/A |
| Imaging Findings | Large herniation compressing cauda equina |
| Decision / Escalation Node | Surgical decompression |

## Supplementary Table S35. Epidural Abscess

| Component | Content |
| --- | --- |
| Clinical Trigger | Back pain, fever, neurological deficits |
| Risk Factors | Diabetes, IV drug use |
| Key Biochemistry | ↑CRP, ↑ESR |
| Imaging Findings | Rim-enhancing epidural collection |
| Decision / Escalation Node | Surgical drainage + antibiotics |

## Supplementary Table S36. Subdural Hematoma

| Component | Content |
|---|---|
| Clinical Trigger | Head trauma, altered consciousness |
| Risk Factors | Elderly, anticoagulants |
| Key Biochemistry | INR abnormalities |
| Imaging Findings | Crescent-shaped extra-axial collection |
| Decision / Escalation Node | Neurosurgical evaluation |

## Supplementary Table S37. Epidural Hematoma

| Component | Content |
|---|---|
| Clinical Trigger | Temporal trauma, LOC |
| Risk Factors | Skull fracture |
| Key Biochemistry | N/A |
| Imaging Findings | Biconvex hyperdense collection |
| Decision / Escalation Node | Emergency surgical evacuation |

## Supplementary Table S38. Cerebral Venous Thrombosis

| Component | Content |
|---|---|
| Clinical Trigger | Headache, seizure |
| Risk Factors | OCP use, hypercoagulability |
| Key Biochemistry | D-dimer (may be ↑) |
| Imaging Findings | Absent flow in dural sinus |
| Decision / Escalation Node | Anticoagulation therapy |

## Supplementary Table S39. Orbital Cellulitis

| Component | Content |
| --- | --- |
| Clinical Trigger | Eye pain, fever, ophthalmoplegia |
| Risk Factors | Sinusitis |
| Key Biochemistry | ↑WBC, ↑CRP |
| Imaging Findings | Orbital fat stranding, abscess |
| Decision / Escalation Node | IV antibiotics ± surgical drainage |

## Supplementary Table S40. Foreign Body Aspiration

| Component | Content |
| --- | --- |
| Clinical Trigger | Sudden cough, wheezing |
| Risk Factors | Young age |
| Key Biochemistry | N/A |
| Imaging Findings | Radiopaque object in airway; air trapping |
| Decision / Escalation Node | Bronchoscopy for removal |
